# How local is the local field potential?

**DOI:** 10.1186/1471-2202-12-S1-O8

**Published:** 2011-07-18

**Authors:** Henrik Lindén, Tom Tetzlaff, Tobias C Potjans, Klas H Pettersen, Sonja Grün, Markus Diesmann, Gaute T Einevoll

**Affiliations:** 1 Dept. of Mathematical Sciences and Technology, Norwegian University of Life Sciences, Ås, 1432, Norway; 2 Institute of Neuroscience and Medicine, Computational and Systems Neuroscience (INM-6), Research Center Jülich, Jülich, 52425, Germany; 3 Brain and Neural Systems Team, RIKEN Computational Science Research Program, Wakoshi, Saitama, 351-0198, Japan; 4 RIKEN Brain Science Institute, Wakoshi, Saitama, 351-0198, Japan

## 

The local field potential (LFP), usually referring to the low-frequency part of an extracellularly recorded potential (< 500 Hz), is nowadays routinely measured together with the spiking activity. The LFP is commonly believed to mainly reflect synaptic activity in a local population surrounding the electrode [1] but how large this population is, i.e. how many neurons contribute to the signal, is still debated. In this modeling study we investigate which factors influence the spatial summation of contributions that generate the LFP signal. A better understanding of this is crucial for a correct interpretation of the LFP, especially when analyzing multiple LFP signals recorded simultaneously at different cortical sites.

We use a simplified two-dimensional model of a cortical population of neurons where the LFP is constructed as a weighted sum of signal contributions from all cells within a certain radial distance to the recording electrode. First we consider a general formulation of the model: if the single-cell LFP contributions can be viewed as current dipole sources [2], the single-cell amplitude will decay as 1/*r*^2^ with distance *r* to the electrode. On the other hand, for the two-dimensional geometry considered here, the number of neurons at a given distance increases linearly with *r*. In addition to these two opposed scaling factors the amplitude of the summed LFP signal also depends on how correlated the single-cell LFP sources are. We calculate the LFP amplitude as a function of the population radius and relate it to the above factors. We show that if the single-cell contributions decay as dipole sources or more steeply with distance, and if the sources are uncorrelated, the LFP is originating from a small local population. Cells outside of this population do not contribute to the LFP. If, however, the different LFP sources are uniformly correlated, cells at any distance contribute substantially to the LFP amplitude. In this case the LFP reach is only limited by the size of the region of correlated sources. This result highlights that the spatial region of the LFP is not fixed; rather it changes with the dynamics of the underlying synaptic activity.

We further validate these results through LFP simulations of morphologically reconstructed cortical cells [2-4] where we study the effects of neuronal morphology on the size of the region contributing to the LFP. Finally, we show the laminar dependence of the reach measure used here and discuss potential implications of the interpretation of experimentally recorded LFPs.
